# Algivore or Phototroph? *Plakobranchus ocellatus* (Gastropoda) Continuously Acquires Kleptoplasts and Nutrition from Multiple Algal Species in Nature

**DOI:** 10.1371/journal.pone.0042024

**Published:** 2012-07-25

**Authors:** Taro Maeda, Euichi Hirose, Yoshito Chikaraishi, Masaru Kawato, Kiyotaka Takishita, Takao Yoshida, Heroen Verbruggen, Jiro Tanaka, Shigeru Shimamura, Yoshihiro Takaki, Masashi Tsuchiya, Kenji Iwai, Tadashi Maruyama

**Affiliations:** 1 Graduate School of Marine Science and Technology, Tokyo University of Marine Science and Technology, 4-5-7, Konan, Minato-ku, Tokyo, Japan; 2 Institute of Biogeosciences, Japan Agency for Marine-Earth Science and Technology, 2-15 Natsushima-cho, Yokosuka, Kanagawa, Japan; 3 Department of Chemistry, Biology and Marine Science, Faculty of Science, University of the Ryukyus, Nishihara-cho, Okinawa, Japan; 4 Phycology Research Group, Ghent University, Ghent, Belgium; 5 School of Botany, The University of Melbourne, Victoria, Australia; 6 Okinawa Prefectural Fisheries and Ocean Research Center, 1-3-1 Nishizaki, Itoman-shi, Okinawa, Japan; University of Melbourne, Australia

## Abstract

The sea slug *Plakobranchus ocellatus* (Sacoglossa, Gastropoda) retains photosynthetically active chloroplasts from ingested algae (functional kleptoplasts) in the epithelial cells of its digestive gland for up to 10 months. While its feeding behavior has not been observed in natural habitats, two hypotheses have been proposed: 1) adult *P. ocellatus* uses kleptoplasts to obtain photosynthates and nutritionally behaves as a photoautotroph without replenishing the kleptoplasts; or 2) it behaves as a mixotroph (photoautotroph and herbivorous consumer) and replenishes kleptoplasts continually or periodically. To address the question of which hypothesis is more likely, we examined the source algae for kleptoplasts and temporal changes in kleptoplast composition and nutritional contribution. By characterizing the temporal diversity of *P. ocellatus* kleptoplasts using *rbcL* sequences, we found that *P. ocellatus* harvests kleptoplasts from at least 8 different siphonous green algal species, that kleptoplasts from more than one species are present in each individual sea slug, and that the kleptoplast composition differs temporally. These results suggest that wild *P. ocellatus* often feed on multiple species of siphonous algae from which they continually obtain fresh chloroplasts. By estimating the trophic position of wild and starved *P. ocellatus* using the stable nitrogen isotopic composition of amino acids, we showed that despite the abundance of kleptoplasts, their photosynthates do not contribute greatly to the nutrition of wild *P. ocellatus*, but that kleptoplast photosynthates form a significant source of nutrition for starved sea slugs. The herbivorous nature of wild *P. ocellatus* is consistent with insights from molecular analyses indicating that kleptoplasts are frequently replenished from ingested algae, leading to the conclusion that natural populations of *P. ocellatus* do not rely on photosynthesis but mainly on the digestion of ingested algae.

## Introduction

Since the discovery of “chloroplast retention” in *Elysia atroviridis* by Kawaguti & Yamasu [1], it has been widely accepted that many species of sacoglossan sea slugs (Sacoglossa, Gastropoda, Mollusca) retain chloroplasts of ingested algae in digestive gland cells [2]. A sequestered chloroplast is called a “kleptoplast” [3]. The kleptoplasts are not passed on to progeny, so new kleptoplasts must be acquired in each generation [4]–[7]. Food algae of sacoglossan species have been studied in laboratory feeding experiments. Most sacoglossan species have a fairly high feeding preference for one or a few algal species [7]–[9], and hence their kleptoplasts come from a limited number of source algae. For example, *Bosellia mimetica* only feed on *Halimeda tuna* [6]–[8] and algae ingested by *Oxynoe antillarum* are limited to 2 species: *Caulerpa racemosa* and *C. sertularioides* [7].

Kleptoplasts retain photosynthetic activity for a few days to several months in some sacoglossan species [10], [11]. Laboratory experiments showed that the photosynthetic products of kleptoplasts (e.g., sugars and amino acids) are transferred to and used by sea slugs when they are starved [12]–[14]. This “functional kleptoplasty” [15] permits sea slugs to have a mixotrophic lifestyle involving photosynthesis via kleptoplasts and heterotrophy by feeding on algae [16]. However, the relative importance of these feeding mechanisms under natural conditions has not been characterized in detail.

The retention period of functional kleptoplasts in the tropical Indo-Pacific species *Plakobranchus ocellatus* van Hasselt, 1824 has been estimated to be up to 10 months from linear extrapolation of the photosynthetic activity measured by pulse amplitude modulation (PAM) fluorometry [10], [17]. The natural algal source species for kleptoplasts in *P. ocellatus* have not been determined [17], [18], but *P. ocellatus* are known to feed on multiple siphonous green algae of the order Bryopsidales under artificial conditions, i.e., *Chlorodesmis hildebrandtii*, *Rhipidosiphon javensis*, *Caulerpella ambigua*, and *Bryopsis* sp. [18]–[20]. It has been proposed that once the sea slug acquires functional kleptoplasts, they are not replenished [21]. This hypothesis is based on the knowledge that the chlorophyll contents of kleptoplasts remain unaffected in starved *P. ocellatus* for 27 days under 500 ft-candle lighting ( = 109.5 µmol photons m^−2^ s^−1^ equivalent of incandescent light) [10], that *P. ocellatus* are mostly found on sandy beaches where potential food algae are rare [21], and that feeding behavior has never been observed in their natural habitats [21]. However, Dunlap [20] reported that the chlorophyll content and ^14^C-inorganic carbon fixation rate diminished after 2 months of incubation under outdoor lighting conditions, and concluded that kleptoplasts in *P. ocellatus* must be replaced continually or at periodic intervals to replace those that have degenerated. These two hypotheses remain to be examined. Before addressing which hypothesis is more likely, a number of questions must be answered. First, which algae are the sources of kleptoplasts for the sea slugs? Second, do kleptoplasts in each *P. ocellatus* individual derive from a single or multiple algal species? Finally, to what degree does the sea slug's nutrition depend on kleptoplast photosynthesis and on algivory?

To address these questions, we identified the source algae of kleptoplasts based on sequences of the RuBisCO (ribulose 1,5-bisphosphate carboxylase/oxygenase) large subunit gene (*rbcL*), which is encoded in the chloroplast genome [9], [22], [23] and has been sequenced for a wide range of siphonous green algae [22]. We assessed the temporal kleptoplast composition in field-collected *P. ocellatus* to determine whether the sea slugs acquire kleptoplasts only once or repeatedly and estimated the trophic positions of the sea slugs in their natural habitat and during starvation in the laboratory based on the nitrogen isotopic composition of glutamic acid and phenylalanine [24], [25], which can be expected to serve as a proxy of the nutritional contribution of functional kleptoplasts to the sea slugs. Based on this combination of techniques, we aimed to gain insight into the ecological role of functional kleptoplasty in natural circumstances.

## Materials and Methods

### Sampling and DNA extraction of *P. ocellatus*

We collected *P. ocellatus* for DNA analyses by snorkeling or scuba diving in a 200-m×200-m near-shore area off Tenija, Okinawa, Japan (26°33′N 128°08′E). No permission was required for sample collection. Samples were taken each month from April to December 2005, and from May 2007 to April 2008. However, the sea slug was seldom found in winter (December 2005 and December 2007 to April 2008). Collected sea slugs were fixed in 99% ethanol at room temperature and preserved at −30°C until use.

With a DNeasy blood and tissue kit (Qiagen, Hilden, Germany), the DNA of *P. ocellatus* was extracted from part of the parapodial tissue (about 5×5×2 mm) (Figure S1), which included the digestive gland (retaining kleptoplasts) but not the stomach [26]. Although a previous study showed chloroplasts on the outside of the stomach cells of *P. ocellatus*, no such “extracellular” chloroplasts were observed in the cavity of the parapodial digestive gland [26], and hence we believe that the sequences obtained did not include contaminant DNA from extracellular chloroplasts in the digestive tract.

### Genetic analysis of *P. ocellatus*

Because it has been proposed that *P. ocellatus* is a cryptic species complex [19], [27], we checked the genetic homogeneity of the collected individuals by sequencing the mitochondrial 16S rRNA gene (mt16S rDNA). To identify the source algae of the kleptoplasts, *rbcL* in 7 individuals of *P. ocellatus* collected in nonconsecutive months were also sequenced. Fragments of *rbcL* and of mt16S rDNA were amplified by PCR with their respective primer sets (Table 1).

**Table 1:** Primer list for sequencing and T-RFLP.

Gene	Primer name	Sequences (5′–3′)	Reference
**16S rDNA** (on *P. ocellatus* (mitochondria)	16sar-F* †	CGC CTG TTT ATC AAA AAC AT	Palumbi et al. [56]
	16sbr-H* †	CCG GTC TGA ACT CAG ATC ACG T	Palumbi et al. [56]
***rbcL*** (on kleptoplasts and chloroplasts)	rbc1* †	CCA MAA ACW GAA ACW AAA GC	Hanyuda et al. [57]
	U3-2* †	TCT TTC CAA ACT TCA CAA GC	Hanyuda et al. [57]
	2 U†	TTG GTW ACW GAA CCT TCT TC	Hanyuda et al. [57]
	rbc5†	GCT TGW GMT TTR TAR ATW GCT TC	Hanyuda et al. [57]
	trbcL-F‡	CTK GCD GYD YTT MGD ATG ACA C	This study
	trbcL-R‡	MRG CWA RWG AAC GTC CTT CAT T	This study

**Primers used for PCR and sequencing of mitochondrial 16S rDNA and kleptoplast (chloroplast)** ***rbcL*** **genes, and for T-RFLP analysis of** ***rbcL*** **.**

* **Primers for PCR.**

† **Primers for sequencing.**

‡ **Primer for T-RFLP.**

The PCR mixture (50 µl) contained 5 µl of 10× Ex Taq Buffer (Takara Bio, Otsu, Japan), 4 µl of dNTP mixture (10 mM), 0.2 µl of TaKaRa Ex Taq (Takara Bio), 1 µl of the template DNA, and 39.8 µl of H_2_O. The thermal cycle was completed under the following conditions: initial denaturation at 96°C for 1 min followed by 35 cycles of 20 s at 96°C, 45 s at 50–55°C, and 1 min 45 s–2 min at 72°C. Amplicons of *rbcL* and mt16S rDNA were purified using a Wizard SV Gel and a PCR Clean-Up System (Promega, Heidelberg, Germany).

Purified amplicons of the *rbcL* genes were cloned using a TOPO TA Cloning Kit with Top 10 *E. coli* (Invitrogen, Carlsbad, CA) according to the manufacturer's instructions. The insert size was checked with PCR, and amplicons with the expected size were purified by Sap/ExoI digestion. The sequencing reactions were performed using a BigDye Terminator v3.1 Cycle Sequencing Kit (Applied Biosystems, Foster City, CA) with the primers used for the PCR experiments and two internal primers (Table 1). The nucleotide sequences were determined with an ABI 3130xl Sequencer (Applied Biosystems). If the genetic distance between two or more *rbcL* sequences was less than 0.001 in *p*-distance, those sequence differences were considered as a PCR error and the minor different sequences were excluded from further analyses. Using the Blastn search and tree reconstruction on partial sequences [28], two chimera sequences were detected and excluded from further processing.

Purified amplicons of sea slug mt16S rDNA were directly sequenced with the primers used for the PCR experiments (Table 1). The sequencing was performed as described above.

### Sampling and genetic analysis of algae

In order to expand the reference dataset of *rbcL* sequences of potential source algae from the study region, species belonging to the siphonous green algal order Bryopsidales were collected at Tenija, Bise (26°42′N 127°52′E), Seragaki (26°30′N 127°52′E), and Maeda (26°26′N 127°45′E) in Okinawa, at Faro de San Rafael (24°18′N 110°20′W) in Mexico, and at Asuncion Island (19°41′N 145°23′E) in the Mariana Islands. No permission was required for sample collection. The samples were fixed in 99% ethanol and kept at −30°C until use. The species sampled were *Rhipidosiphon lewmanomontiae*, *Rhipidosiphon* sp., *Caulerpa subserrata*, *Halimeda borneensis*, *Chlorodesmis fastigiata*, and *Poropsis* sp.

We pressed voucher specimens from part of each sample and deposited them in the Herbarium of the Coastal Branch of the Natural History Museum and Institute, Chiba (CMNH) (specimen numbers CMNH-BA-6809–6816), Japan, or in the Ghent Herbarium, Belgium (G.008 and HV1774). The DNA was extracted from the ethanol-fixed algal tissues with a DNeasy blood and tissue kit (Qiagen), an Isoplant kit (Nippon Gene, Toyama, Japan), or a DNeasy plant kit (Qiagen). Then, algal *rbcL* genes were amplified by PCR and directly sequenced with the same primers used for PCR (Table 1) as described above.

### Phylogenetic analyses of kleptoplast *rbcL*

The *rbcL* sequences obtained from *P. ocellatus* and algae were aligned with those of Bryopsidales and Dasycladales species in the DNA Data Bank of Japan (DDBJ) using MAFFT version 6.818b [29] with the “—auto” option. Ambiguously aligned sites were removed automatically using trimAl version 1.2 [30] with the “-automated1” option. The multiple sequence alignment finally showed 1254 positions. Phylogenetic analyses were performed with MEGA5 [31] for the neighbor-joining (NJ) method, and with RAxML version 7.2.8 [32] for the maximum likelihood (ML) method. For the NJ method, the maximum composite likelihood method for nucleotide sequences [33] was employed. Kakusan4 [34] was used to select the appropriate model of nucleotide evolution for ML analysis, and the general time-reversible model [35] incorporating among-site rate variation approximated by a discrete gamma distribution with 4 categories (GTR+Γ) was chosen. The nonparametric bootstrap was used to examine the robustness of phylogenetic relationships (1000 pseudoreplicates for NJ and 300 for ML).

### Terminal restriction fragment-length polymorphism analysis of *rbcL* from *P. ocellatus*

We performed terminal restriction fragment-length polymorphism (T-RFLP) analysis [36] to assess the diversity of kleptoplasts in the sea slugs and the seasonality of their relative abundance. To avoid the biases of the amplification efficiency of PCR due to the high/low matching of the primers, we newly designed nested consensus primers for T-RFLP based on the *rbcL* sequences obtained (Table 1). The primers were designed to amplify the fragment including a specific restriction site for distinguishing different source algae of the kleptoplasts. We digested the *rbcL* regions obtained from *P. ocellatus* with various restriction enzymes *in silico* using TRiFLe [37]. *Tai*I (Thermo Fisher Scientific, Waltham, MA) was found to be the most suitable to identify the multiple-source algal species of kleptoplasts (Table 2).

**Table 2:** *In silico* T-RF lengths of *rbcL* sequences.

Clade in Figure 1	Predicted T-RF length *in silico* (bp)	T-RFLP-category§	Abbreviation (clade)
Clade A (*Proposis* spp.)*	306	*Proposis* spp./*Halimeda borneensis*	Prsp/Habo (A/D)
Clade B (*Rhipidosiphon lewmanomontiae*)	331	*Rhipidosiphon lewmanomontiae*	Rhle (B)
Clade C (*Rhipidosiphon* spp.)	366	*Rhipidosiphon* spp.	Rhsp (C)
Clade D (*Halimeda borneensis*)*	306	*Proposis* spp./*Halimeda borneensis*	Prsp/Habo (A/D)
Clade E (Halimedineae spp. 1)†	138	Halimedineae spp. 1/Rhipiliaceae spp.	Hasp1/Risp (E/G)
Clade F (*Caulerpella* spp.)‡	191/291	*Caulerpella* spp.	Casp (F)
Clade G (Rhipiliaceae spp.)†	138	Halimedineae spp. 1/Rhipiliaceae spp.	Hasp1/Risp (E/G)
Clade H (Halimedineae spp. 2)	260	Halimedineae spp. 2	Hasp2 (H)

* T-RF lengths of *Proposis* spp. and *Halimeda borneensis* were the same and indistinguishable.

† T-RF lengths of Halimedineae spp. 1 and Rhipiliaceae spp. were identical and indistinguishable.

‡ *Caulerpella* spp. was composed of two subtypes having two distinct T-RFs (191 and 291 bp, respectively).

§ See Figures 3 and 4.

The *rbcL* fragments were amplified from the DNA templates of *P. ocellatus* by PCR with the fluoresceinated primer trbcL-F and the nonfluoresceinated primer trbcL-R (Table 1). Thermal cycling was performed under the following conditions: initial denaturation at 96°C for 1 min followed by 35 cycles of 20 s at 96°C, 45 s at 57°C, and 1 min at 72°C. The composition of the PCR mixture was the same as that for clone sequencing. Amplicons in the reaction mixtures (50 µl) were purified with a Wizard SV Gel and PCR Clean-Up System (Promega, Madison, WI). The purified amplicons were digested with 0.5 µl of FastDigest *Tai*I (Fermentas, Vilnius, Lithuania) and then 0.5 µl of 10× FastDigest Buffer (Fermentas) in a total volume of 5 µl at 65°C for 15 min. The digestion product was mixed with 9 µl of Hi-Di formamide (Applied Biosystems) and 0.5 µl of GS 1200 (Liz) (Applied Biosystems) and then denatured by heating at 95°C for 2 min. The mixture was immediately chilled on ice and then electrophoresed with an ABI 3730xl Sequencer (Applied Biosystems).

T-RFLP profiles were analyzed using GeneMapper ver. 3.7 (Applied Biosystems). The internal size standards in electrophoresis gave an indication of the length of the terminal restriction fragments (T-RFs) obtained. Based on the different lengths (base pairs [bp]) of T-RFs, the source algae of the kleptoplasts were distinguished. Relative amounts of the respective *rbcL* sequences were estimated from the heights of T-RF peaks in T-RFLP electropherograms [36], [38].

Chromatograms with high-quality value (QV>75) were selected for further analyses. Fragments smaller than 100 bp and larger than 1200 bp were considered noise and excluded from further analyses. Each T-RF was identified by matching to the *in silico* digestion lengths of *rbcL* sequences from kleptoplasts (Table 2). The relative abundance was calculated from relative peak heights using the formula RA (%) = H_T-RF_/H_total_*100 (where RA is relative abundance, H_T-RF_ is the peak height of a specific T-RF, and H_total_ is the total of all T-RF peak heights in each chromatogram). The statistical significance of the differences in *rbcL* abundance between seasonally collected samples was assessed with the permuted Brunner-Munzel test [39] implemented in the “lawstat” package for “R” [40].

### Amino acid nitrogen isotopic analysis

To estimate the trophic position of the sea slugs under natural conditions and during starvation, we used amino acid nitrogen isotopic analysis [24], [25]. The individuals of *P. ocellatus* for the amino acid nitrogen isotopic analysis were collected off Toguchi, Okinawa, Japan (26°21′N 127°44′E). No permission was required for sample collection. About 50 individuals were collected on 27 April 2010. For transport to the laboratory in Kanagawa, Japan, they were kept alive in artificial seawater (Rhotomarine, Rei-Sea, Tokyo, Japan) at room temperature for 3 days without lighting. Three healthy individuals were randomly selected and dissected, and parapodial tissues were frozen and kept in liquid nitrogen as wild samples. The samples for starvation were collected on 8 April 2008 (about 50 individuals) and incubated in an aquarium filled with artificial seawater not containing macroalgae at 24°C under light (13 µmol photons m^−2^ s^−1^) with day–night (14-h light/10-h dark) rhythms. After incubation for 156 days (about 5 months), 3 individuals were randomly selected and dissected. Their parapodial tissues were also frozen and kept in liquid nitrogen until analysis.

The nitrogen isotope analysis of amino acids was performed according to the method of Chikaraishi *et al.* [25]. Part of the frozen parapodial tissue of each *P. ocellatus* individual was hydrolyzed in 12N HCl at 100°C. The hydrolysate was washed with *n*-hexane/dichloromethane (6∶5, v/v) to remove any hydrophobic constituents. After derivatization with thionyl chloride/2-propanol (1∶4, v/v) and subsequently with pivaloyl chloride/dichloromethane (1∶4, v/v), the derivatives of the amino acids were extracted with *n*-hexane/dichloromethane (6∶5, v/v). The nitrogen isotopic composition of each amino acid was determined by gas chromatography/combustion/isotope ratio mass spectrometry using a Delta plus XP isotope ratio mass spectrometer (Thermo Finnigan MAT, Bremen, Germany) coupled to an 6890N gas chromatograph (Agilent Technologies, Massy, France) via combustion and reduction furnaces. Nitrogen isotopic compositions were expressed in δ-notation against atmospheric N_2_ (air). The trophic position of the organism was calculated from the nitrogen isotopic ratio in glutamic acid and phenylalanine (TP_Glu/Phe_ value) with the formula TP_Glu/Phe_ = (δ^15^N_Glu_−δ^15^N_Phe_−3.4)/7.6+1 [24].

We also determined the TP_Glu/Phe_ value of a giant clam, *Tridacna crocea*, harboring a symbiotic dinoflagellate, zooxanthellae (*Symbiodinium* spp.) [41], and of an identified kleptoplast source alga, *R. lewmanomontiae*. Three individuals of *T. crocea* were collected off Sesokojima, Okinawa (26°38′N 127°52′E) on 19 November 2011. After 3 days of incubation without feeding and light for transport to the laboratory, the mantle tissues containing zooxanthellae and adductor muscles without zooxanthellae were dissected from each individual. The adductor muscles were analyzed using the same method as for the tissue of *P. ocellatus*. To isolate zooxanthellae, mantles of each individual were cut into pieces, homogenized with a polytron-type homogenizer (T-10 basic homogenizer, IKA, Staufen, Germany) with 20 ml of artificial seawater, and centrifuged at 750×*g* for 3 min [42]. After decantation of the supernatant, the pellet was suspended with 20 ml of artificial seawater. The suspension was filtered through a 20-µm mesh nylon cloth to remove host tissue debris and washed two times with 20 ml of artificial seawater. Pelleted zooxanthellae were used for the amino acid nitrogen isotopic analysis. A thallus of *R. lewmanomontiae* was collected at the same site with *P. ocellatus* (off Toguchi, Okinawa) on 27 April 2010 and its δ^15^N was analyzed as described above.

## Results

### Identification of *P. ocellatus* kleptoplasts

To confirm the genetic homogeneity of *P. ocellatus* collected and identify the source algae of kleptoplasts, we sequenced their mt16S rDNA and *rbcL*. The sequences of mt16S rDNA fragments [443 bp: DNA Data Bank of Japan (DDBJ) accession number, AB700359] were identical among all examined *P. ocellatus* individuals collected off Tenija, Okinawa, Japan, suggesting that all samples belonged to the same species.

By clone sequencing, we obtained 209 *rbcL* sequences of kleptoplasts from 7 individuals of *P. ocellatus* collected in 2005 (Table 3). After unifying highly similar sequences (*p*-distance<0.001), 59 sequences were used for subsequent analyses (DDBJ accession numbers, AB619256–AB619314).

**Table 3:** Number of clones obtained from kleptoplast *rbcL* clone sequences.

	Corresponding clades (in Figures 1 and 2) of clones								Total
	A	B	C	D	E	F	G	H	
ID of the individual (month of collection)									
Po-2005-A (Apr.)	1	-	-	-	7	-	-	8	16
Po-2005-B (May)	18	-	1	-	2	-	-	-	21
Po-2005-C (June)	7	5	4	-	-	3	-	-	19
Po-2005-D (Aug.)	10	3	4	-	-	7	-	-	24
Po-2005-E (Aug.)	82	-	-	1	-	-	5	2	90
Po-2005-F (Sept.)	11	-	-	-	-	4	-	-	15
Po-2005-G (Nov.)	13	-	4	1	-	6	-	-	24
Total number of clones	142	8	13	2	9	20	5	10	209

**Figure 1:**
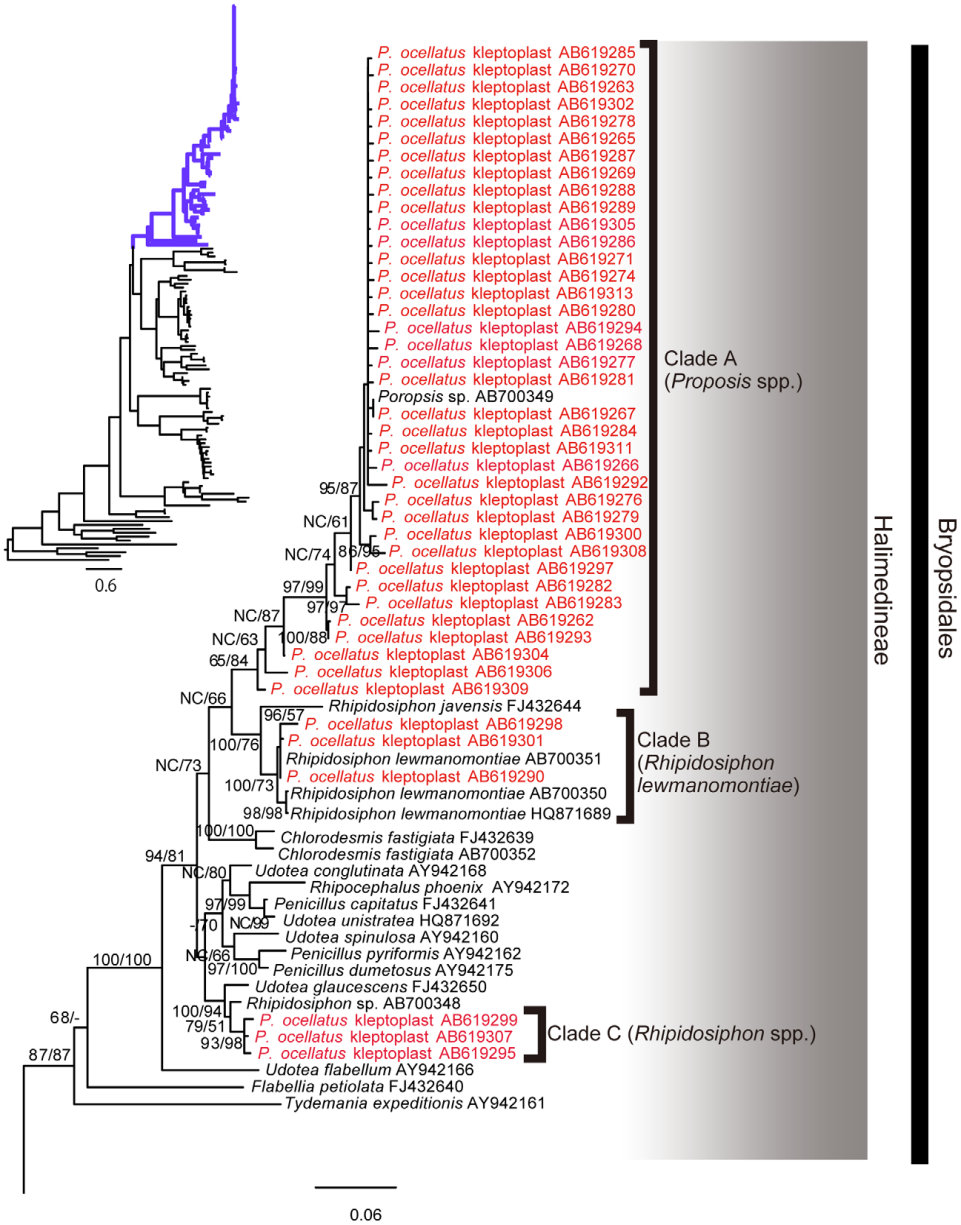
Phylogenetic tree based on *rbcL* sequences. Maximum likelihood (ML) phylogeny of the class Ulvophyceae based on 1254 nucleotide positions of the chloroplast-encoded *rbcL* gene. The phylogram of the entire tree on the upper left is colored to match the inset. *Chlamydomonas reinhardtii* (class Chlorophyceae) was chosen as the outgroup. Red, *rbcL* sequences from *P. ocellatus*. Black, algal *rbcL* sequences. Bootstrap support (BS) values >50% are provided at the nodes (neighbor-joining/ML). “-“, BS <50%; “NC”, nonconsensus node that was not observed in the neighbor-joining tree.

Number of clones obtained in kleptoplast *rbcL* clone sequencing from each of 7 *Plakobranchus ocellatus* individuals. The source algae were identified from the phylogenetic analysis (Figure 1).

**Figure 2:**
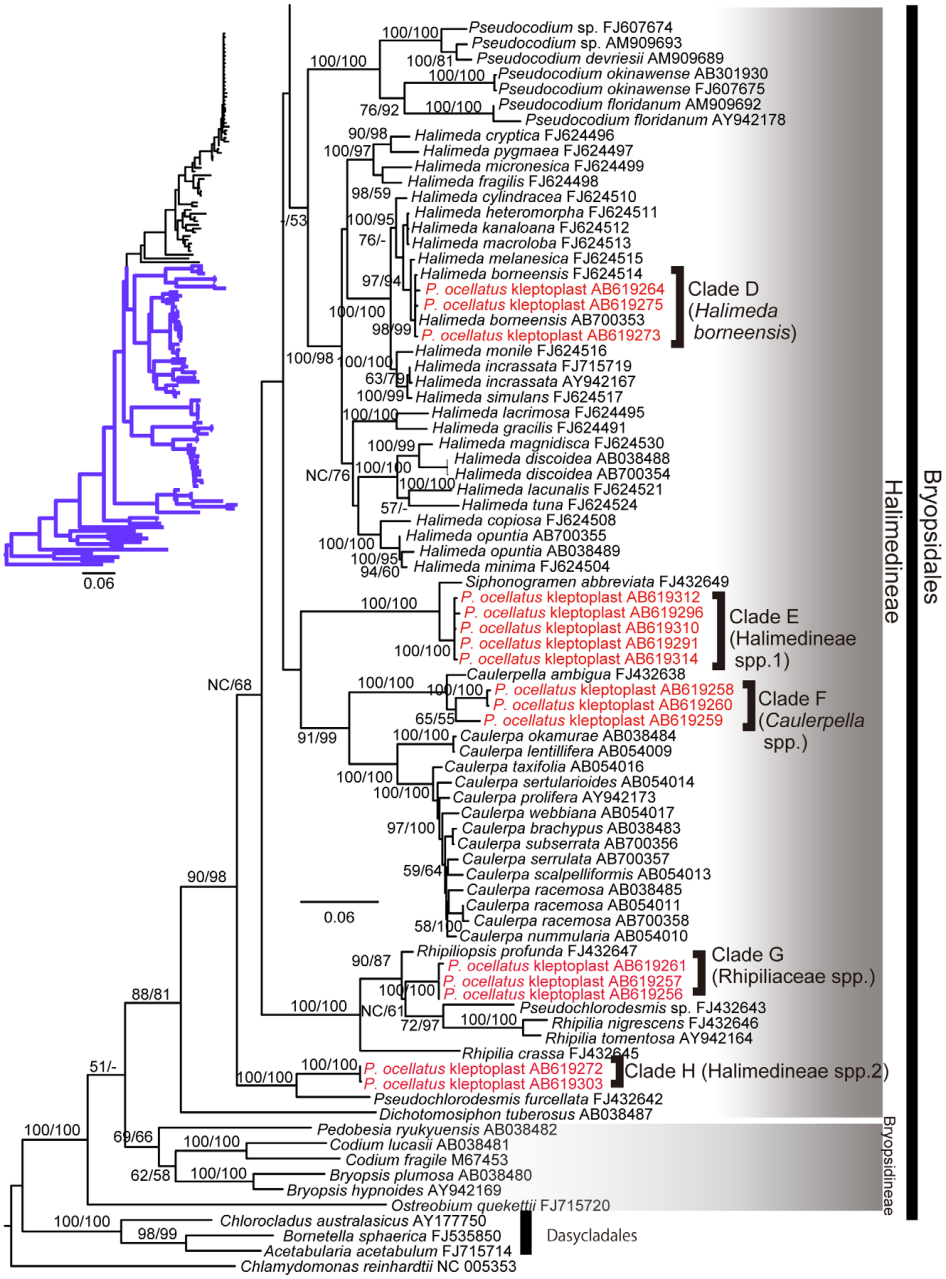
Phylogenetic tree based on *rbcL* sequences (continued).

Blastn analyses showed that the kleptoplast *rbcL* sequences were the most similar to those from green algae belonging to the suborder Halimedineae (order Bryopsidales). In the phylogenetic tree (Figures 1 and 2), the kleptoplast sequences did not form a monophyletic clade but were divided into eight clades within the Halimedineae (clades A–H in Figures 1 and 2). Clade B sequence AB619290 was identical to *R. lewmanomontiae* from Tenija (AB700351). Clade D corresponded to reference sequences of *H. borneensis* (FJ624514 and AB700353). The remaining 6 clades of kleptoplasts (A, C, E, F, G, H) could not be identified at the species level and were assigned to higher taxa. Clade A clustered with *Poropsis* sp., clade C with *Rhipidosiphon* sp., clade F with the genus *Caulerpella*, and clade G with the family Rhipiliaceae. Familial relationships were unclear for clades E and H, but they both belong to the higher taxon Halimedineae. For convenience, the source algae with such imperfect identifications are indicated with a higher taxon name+spp. (e.g., Rhipiliaceae spp.).

Based on the *rbcL* sequences cloned from the 7 individuals, it was already clear that individual sea slugs contained kleptoplasts from multiple source species. As shown in Table 3, no single individual had only one type of kleptoplast. In most cases, kleptoplasts from 3 or 4 algal species were present in *P. ocellatus* individuals.

### Seasonality of *P. ocellatus* kleptoplasts

To examine whether the composition of kleptoplasts changed over time, T-RFLP analysis of *rbcL* with *Tai*I digestion was performed. Halimedineae spp. 1 (clade E) and Rhipiliaceae spp. (clade G) could not be distinguished in this analysis because their predicted T-RF lengths were identical at 138 bp (Table 2). They are hereafter referred to as “Hasp1/Risp (clade E/G).” The T-RF of *Poropsis* spp. (clade A) could not be differentiated from that of *H. borneensis* (clade D) (T-RF length = 306 bp, Table 2), and they are referred to as “Prsp/Habo (clade A/D).” The heights of those T-RF peaks were regarded as the sum of the respective two algal source clades (Table 2).

**Figure 3:**
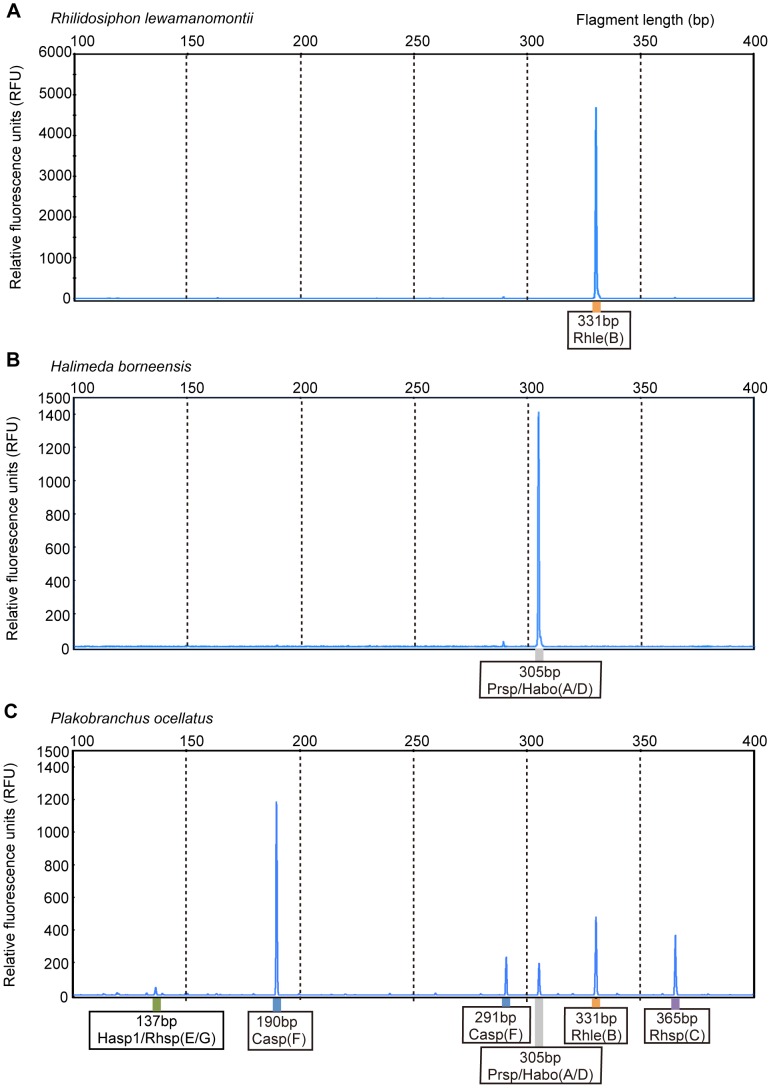
Electropherograms of T-RFLP analysis. T-RFLP electropherograms from (A) *Rhipidosiphon lewmanomontiae*, (B) *Halimeda borneensis*, and (C) a single individual of *Plakobranchus ocellatus* (sample no. Ploc051113A). Fragment lengths and corresponding source algae are shown in the box under the peak. Only a single peak with a length identical to that predicted by *in silico* T-RFLP was obtained from each algal individual (Table 2). The electropherogram from the sea slug (C) showed multiple peaks corresponding to those of *in silico* T-RFLP of the kleptoplast source algae (Table 2).

To establish the applicability of *in silico* T-RF predictions in actual T-RFLP analysis, we compared the predicted T-RF fragment lengths with those of PCR-amplified *rbcL* fragments from two algae, *H. borneensis* and *R. lewmanomontiae*. We obtained a single T-RF peak in each algal species, and its T-RF length was identical with that predicted *in silico* (*H. borneensis* = 306 bp and *R. lewmanomontiae* = 331 bp) (Table 2, Figure 3A, B).

**Figure 4:**
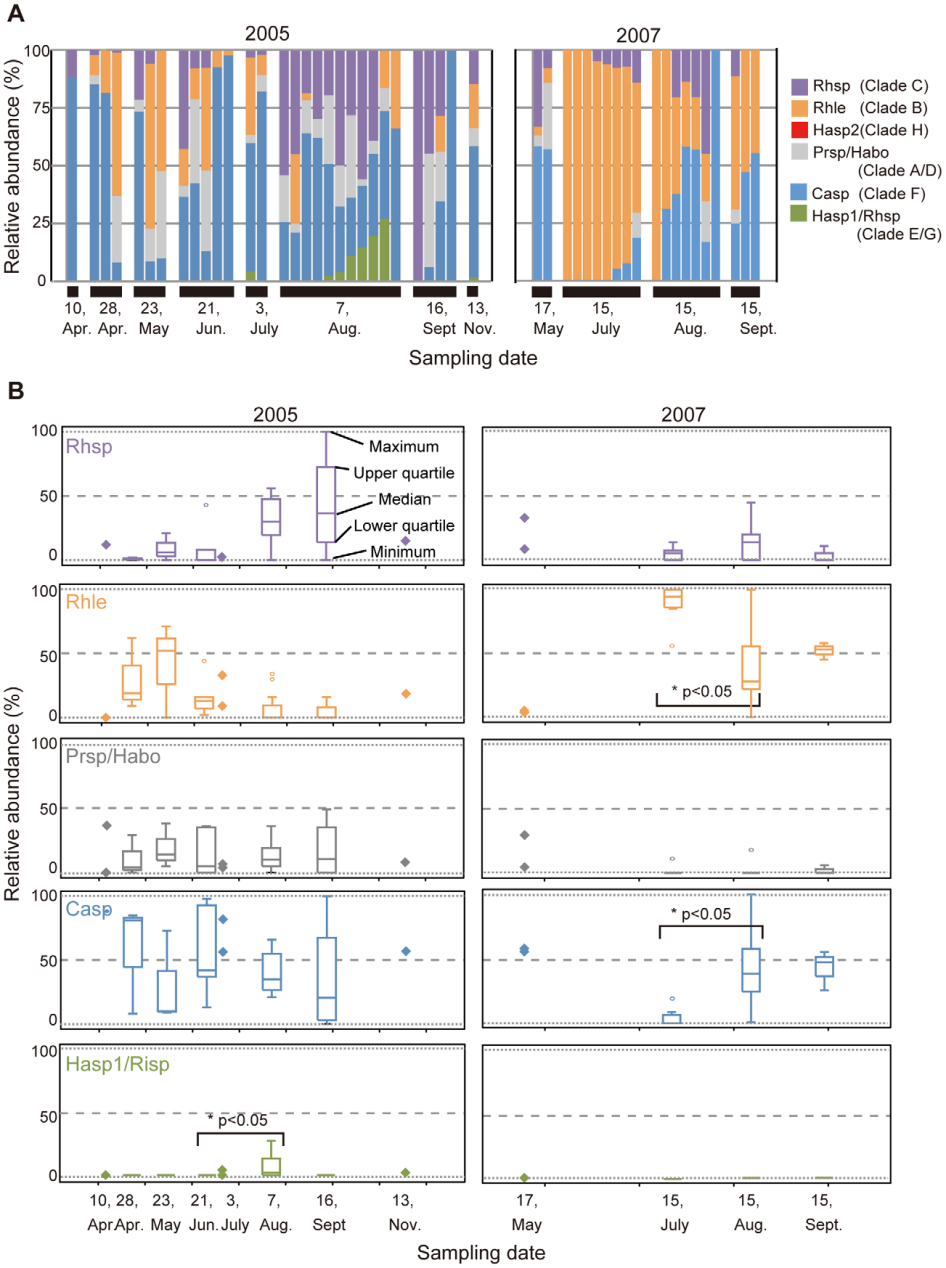
Time series of *rbcL* sequence composition in *Plakobranchus ocellatus*. (A) Relative abundance of respective *rbcL* in individuals of *Plakobranchus ocellatus* collected monthly in 2005 and 2007. Source algae of kleptoplasts are shown by abbreviations of the clades (Table 2). (B) Tukey's boxplots of the relative abundance of *rbcL* sequences. Data points of fewer than three individuals of *P. ocellatus* are shown as diamonds. The brackets with “*” indicate a significant difference of the percentage value in the permuted Brunner-Munzel test. The value beside the symbol “*” indicates p-value.

High-quality T-RFLP electropherograms (quality value >75) were obtained from 30 individuals of *P. ocellatus* collected in 2005 and from 20 collected in 2007 (Figure 4A). One to five peaks were found in each *P. ocellatus* individual (Figures 3C and 4A, Table S1). Except for the peak corresponding to Halimedineae spp. 2 (clade H) (260 bp), all predicted restriction profiles were recovered. Forty-four out of 50 individuals of *P. ocellatus* showed multiple T-RF peaks (28 individuals in 2005 and 16 in 2007) (Figure 4A, Table S1).

Assuming that the genome copy number in the chloroplasts did not change before and after sequestration, the relative abundance of *rbcL*s was used to estimate that of the corresponding kleptoplasts. As shown by the boxplots in Figure 4B, the relative abundance of kleptoplasts showed marked seasonal changes. Some of the observed changes occurred over a relatively short time-span. For example, in 2005, the abundance of Hasp1/Risp (clade E/G) in August was significantly higher than that in June (p-value = 0.025) (Figure 4B). Similarly, in 2007, the percentage of *Caulerpella* spp. (clade F) significantly increased from July to August (p-value = 0.010), while that of *R. lewmanomontiae* (clade B) significantly decreased in the same period (p-value = 0.020) (Figure 4B). In 2005, clear changes in abundance were also observed for *Rhipidosiphon* spp. (clade C), *R. lewmanomontiae* (clade B), and Hasp1/Risp (clade E/G) but these were not significant in the permuted Brunner-Munzel test (p-value = 0.097, 0.571, and 0.102, respectively) (Figure 4B).

### Trophic position of *P. ocellatus*

To estimate the trophic position of *P. ocellatus*, the δ^15^N values of glutamic acid (δ^15^N_Glu_) and phenylalanine (δ^15^N_Phe_) of the freshly collected and starved samples of *P. ocellatus* were measured, and TP_Glu/Phe_ values were calculated. The mean TP_Glu/Phe_ value of 3 freshly collected *P. ocellatus* individuals was 1.9±0.1 (mean ± “1σ for the TP values,” *n* = 3) (Table 4). On the other hand, the mean TP_Glu/Phe_ values of *P. ocellatus* that had been starved under light for about 5 months (156 days) was 1.3±0.1 (*n* = 3) (Table 4). The TP_Glu/Phe_ value of *R. lewmanomontiae*, which was a source alga of kleptoplasts in *P. ocellatus*, was 1.0 (*n* = 1). The mean TP_Glu/Phe_ value of *T. crocea* adductor muscle was 2.0±0.1 (*n* = 3), and that of zooxanthellae isolated from *T. crocea* mantle was 0.9±0.0 (*n* = 3).

**Table 4:** δ^15^N values of amino acids and estimated trophic positions of *Plakobranchus ocellatus*, the alga *Rhipidosiphon lewmanomontiae*, and the giant clam *Tridacna crocea*.

Specimen	Number of specimens	Averaged δ^15^N value§ (SD)		Trophic position#
		Glutamic acid	Phenylalanine	
*Plakobranchus ocellatus*				
Freshly collected* (Apr. 2010)	3	17.4 (0.9)	7.3 (1.3)	**1.9 (0.08)**
Starved† (Apr.–Nov. 2008)	3	20.0 (0.5)	14.4 (0.9)	**1.3 (0.07)**
*Rhipidosiphon lewmanomontiae* (Apr. 2010)	1	12.3	8.8	**1.0**
*Tridacna crocea* (Nov. 2011)				
Adductor muscle	3	16.1 (0.6)	5.5 (0.1)	**2.0 (0.07)**
Zooxanthellae‡	3	8.2 (0.4)	5.3 (0.1)	**0.9 (0.04)**

* Collected off Toguchi, Okinawa, Japan.

† Starved for 156 days (5 months) in a laboratory aquarium after collection off Toguchi.

‡ *Symbiodinium* spp. isolated from the mantle of *T. crocea*.

§ ‰, relative to air.

# Trophic position (TP_Glu/Phe_) = (δ^15^N_Glu_−δ^15^N_Phe_−3.4)/7.6+1.

## Discussion

By combining different methods, our results offer several new insights into the ecology of functional kleptoplasty in *P. ocellatus*. Because unincubated wild individuals were studied, the results shed light on the natural state of this sea slug species.

### Multiple algal sources of kleptoplasts in *P. ocellatus*

The phylogenetic analyses of the chloroplast-encoded *rbcL* gene identified at least 8 algal sources of kleptoplasts in *P. ocellatus* (Figure 1). This number exceeds the estimates from previous laboratory feeding experiments on *P. ocellatus* [18], [20] Our data demonstrate that *P. ocellatus* has one of the broadest food algal spectra in the Sacoglossa, including at least 8 species in 5 different algal genera [7]. The multiplicity of kleptoplasts in individual sea slug specimens also confirms that individuals have multiple food sources. This multiplicity of kleptoplasts was first reported in a sacoglossan, *Elysia clarki* (2–4 species) [9], and also recently suggested in *P. ocellatus* [17]. These findings strongly suggest that, in the natural setting, the sea slugs feed on several species and retain their chloroplasts in digestive gland cells.

A common conclusion of our work and previous feeding studies on *P. ocellatus* [7], [18], [20] is that their food/source algae are all siphonous green algae of the order Bryopsidales. The genus *Caulerpella*, which was identified as a food source in a previous study [20], was shown to be a major contributor to the kleptoplast pool. On the other hand, we did not detect 3 algal species that were eaten by *P. ocellatus* in a laboratory setting in previous studies: *C. hildebrandtii* (Udoteaceae); *Bryopsis* sp.; and *R. javensis* [18], [20]. Such discordance may be because the algae in question are not preferred by *P. ocellatus* in their natural habitats, or because the algae are ingested but their chloroplasts are not retained. For some of the algal species involved, misidentifications could also lead to the mismatch. For example, *Rhipidosiphon* is a genus of diminutive, fan-shaped algae in which there are several morphologically similar but genetically distinct species (Verbruggen, unpublished results), and kleptoplast clade C may originate from algae that closely resemble *R. javensis*. Similarly, it is possible that in the previous studies the source algae belonging to clade A (genus *Poropsis*) could have been misidentified as *C. hildebrandtii*. Because these taxa are anatomically very similar, and because the genus *Poropsis* is little known and not commonly included in taxonomic reference works, it may have been regarded as *C. hildebrandtii* in previous studies.

Our kleptoplast survey also revealed several new food algae of *P. ocellatus*. Among them are *R. lewmanomontiae* (clade B), *H. borneensi*s (clade D), Rhipiliaceae spp. (clade G), and potentially *Rhipidosiphon* spp. (clade B). In addition to these taxa, we found kleptoplasts of two lineages that could not be readily assigned to a family (Halimedineae spp. 1 and spp. 2). The most closely related *rbcL* sequences of these two clades are *Pseudochlorodesmis* species (Figure 1). The diminutive siphon of *Pseudochlorodesmis*, if branched at all, does so only a few times [43]. Because the algae rarely exceed a few millimeters in length, it should not come as a surprise that they may have been overlooked in previous studies. The observed ulvophyceaen macroalgae in sampling sites are listed in Table S3.

An interesting observation is that *P. ocellatus* do not seem to discriminate among food sources based on size. Among the sources of kleptoplasts are some larger seaweeds such as *H. borneensis* (10 cm) and *R. lewmanomontiae* (3 cm) as well as several millimeter-scale taxa (*Pseudochlorodesmis*, *Caulerpella*). One commonality between these algae is that they all belong to suborder Halimedineae of the order Bryopsidales (Figure 1) [44], which is consistent with the hypothesis on the relationships between the radular tooth shape and food algal cell wall components [45]. The radular tooth of *P. ocellatus* has a triangular shape suitable for puncturing a hole in the halimedineaen cell wall, which is composed of xylan [45], [46], after which the cytoplasm can be ingested.

### Changing annual kleptoplast composition

T-RFLP analysis showed that the kleptoplast *rbcL* composition of the sea slugs changed considerably from month to month. Because kleptoplasts have not been observed to divide [2], [17], these changes must result from the replacement of kleptoplasts with newly obtained chloroplasts by feeding, different spans of longevity among kleptoplasts derived from various source algae, and/or differential kleptoplast DNA replication (multimerization) rates [47], [48] due to those source algae.

Different spans of longevity and change in chloroplast genome multimerizations of the kleptoplasts would leave a specific imprint on the kleptoplast composition over time, i.e., the recovered kleptoplast composition would remain the same but their relative abundance should increase/decrease gradually over time. Our results do not suggest such a pattern but rather suggest that large, apparently stochastic changes occur in kleptoplast composition. Although we cannot entirely exclude the possibility that *P. ocellatus* individuals immigrated into the population at the collection sites, previous studies suggested that *P. ocellatus* breed in a restricted season in spring and rarely migrate as adults [18], [49]. Therefore, the hypothesis that kleptoplasts are replaced with newly obtained chloroplasts from ingested algae appears to be the most plausible explanation for our results. Although the seasonal change in the algal community may affect the kleptoplast compositions in *P. ocellatus*, this point remains to be studied in future.

### Trophic position of *P. ocellatus*

Assuming an error in the TP_Glu/Phe_ value (i.e., 1σ = 0.12) [24], the values of wild *P. ocellatus* (mean TP_Glu/Phe_ value = 1.9) were identical to the trophic position of algivorous organisms, i.e., primary consumers (Table 4, S2). These results clearly indicate that, under natural circumstances, *P. ocellatus* individuals mainly obtain amino acids by digestion of ingested algae. This is in stark contrast to *P. ocellatus* individuals that had been starved for 5 months and had a TP_Glu/Phe_ value of 1.3, which is intermediate between values typical of phototrophic organisms (primary producers) and algivores (primary consumers).

These results suggest that kleptoplasts of *P. ocellatus* could produce amino acids, for which the TP_Glu/Phe_ value would be 1.0, similar to that of primary producers. The production of amino acids from inorganic nitrogen was also reported in kleptoplasts of *E. viridis* in a tracer experiment using ^15^N-inorganic nitrogen [14]. Teugels and coworkers have proposed that kleptoplasts synthesize glutamic acid from ammonium derived from seawater or made from nitrate and/or urea, and that other amino acids are synthesized from glutamic acid [14]. It is not clear whether the enzymes of these pathways remain active in the kleptoplasts [17] or whether their genes are expressed in the host animal nucleus to which they were transferred from the algal nuclear genome [50]. When the amount of amino acids from the digestion of algae is much larger than that from kleptoplasts, the TP_Glu/Phe_ value would become 2.0. Accordingly, the observed TP_Glu/Phe_ values (i.e., 1.3±0.1) for cultured individuals imply some significant contribution of the kleptoplast-produced amino acids to *P. ocellatus* during starvation.

The giant clam *T. crocea* harbors zooxanthellae, which contribute significantly to the host nutrition, but it also feeds on free-living plankters [51]. Previous studies showed that the zooxanthellae secrete a photosynthate, glycerol or glucose, in the mantle tissue [42], [52], [53] but the zooxanthellae are also digested in the stomach [54], [55]. Those previous results suggested that essential amino acids are derived from the digestion of zooxanthellae and plankters. The TP_Glu/Phe_ value of the giant clam *T. crocea* was 2.0, which is in agreement with previous results [54], [55] showing that *T. crocea* gain amino acids by the digestion of zooxanthellae in symbiotic relationships as well as the digestion of free-living plankters. The data together with those of natural and starved individuals suggest that wild *P. ocellatus* obtain amino acids mainly from heterotrophic digestion of algae as in the case of *T. crocea*, while starved individuals obtain significant amounts of amino acids from photosynthetic production in kleptoplasts.

### Feeding of *P. ocellatus* under natural conditions

The present results indicate that *P. ocellatus* gain kleptoplasts from multiple algal species, that each individual harbors a heterogeneous population of kleptoplasts from multiple algal species, that the kleptoplast composition changes with time, and that the sea slugs mainly gain amino acids by digesting ingested algae in nature, although kleptoplasts are probably capable of producing amino acids. These combined findings suggest that wild *P. ocellatus* repeatedly feed on siphonous green algae and acquire new kleptoplasts when there is sufficient algal biomass to feed the *P. ocellatus* population. The ecological role of functional kleptoplasty has been hypothesized to be a nutrition resource during periods of food insecurity [6], [15]. The photosynthetic activity of kleptoplasts in starved *P. ocellatus* is reduced by about 10% per month [10]. Replenishing kleptoplasts equivalent to the photosynthetic activity lost is necessary to sustain the required level of photosynthetic activity.

Although *P. ocellatus* is capable of retaining kleptoplasts for up to 10 months, our results indicate that the nutritional contribution of kleptoplasts may be insufficient even in a natural habitat where food algae are plentiful. In other sacoglossan species such as *Elysia timida*, *E. viridis*, and *Thuridilla carlsoni*, the nutritional contribution of functional kleptoplasts may be even less, because the photosynthetic activity of their kleptoplasts is less and retention periods are shorter than in *P. ocellatus* [10]. The ecological role of kleptoplasts under natural conditions when food is abundant remains unclear. Functional kleptoplasty may reduce the algal feeding and the energy cost to ingest their calcified thallus [6]. Our present study showed that the nutritional yield from kleptoplasts does not exceed that from food digestion but suggested that they provide an energy source (e.g., sugars) and essential nutrients (e.g., amino acids) under starved conditions. The TP_Glu/Phe_ value of 1.3 during starvation suggests that the contribution of kleptoplasts to amino acid synthesis is much greater than that of autophagic digestion of reserved amino acids and proteins, which would increase the TP_Glu/Phe_ value. It has been proposed that the function of kleptoplasts may be to complement nutrients under starved conditions [6]. Our present results support this hypothesis.

Although adult *P. ocellatus* are thought to be nourished by the photosynthetic activity of functional kleptoplasty, the present study suggests that the sea slug continually feeds on siphonous green macroalgae in its natural habitat and that digested algae are the major source of nutrition, while kleptoplast photosynthesis plays a very minor role. Further studies on the feeding behavior, photosynthetic activity, and nutritional contribution of kleptoplasts in *P. ocellatus* are necessary to understand the ecological role of the functional kleptoplasty of this species.

## Supporting Information

Figure S1 **Overviews of** ***Plankobranchus ocellatus*** **.** (A) Dorsal views of a freshly collected intact *P. ocellatus* individual. (B) An anesthetized individual with spread parapodia. The tissue region in the red square was dissected and used for DNA extraction.
(TIF)

Table S1 **Percentage abundance of** ***rbcL*** **sequences from source algae in** ***P. ocellatus*** *.
(XLS)

Table S2 (XLS)

Table S3 (XLS)
