# Supplementary figures and images for: Algivore or Phototroph? Plakobranchus ocellatus (Gastropoda) Continuously Acquires Kleptoplasts and Nutrition from Multiple Algal Species in Nature
Source: PLoS One. 2012 Jul 25;7(7):e42024. doi: 10.1371/journal.pone.0042024 (PMC3404988; doi:10.1371/journal.pone.0042024)

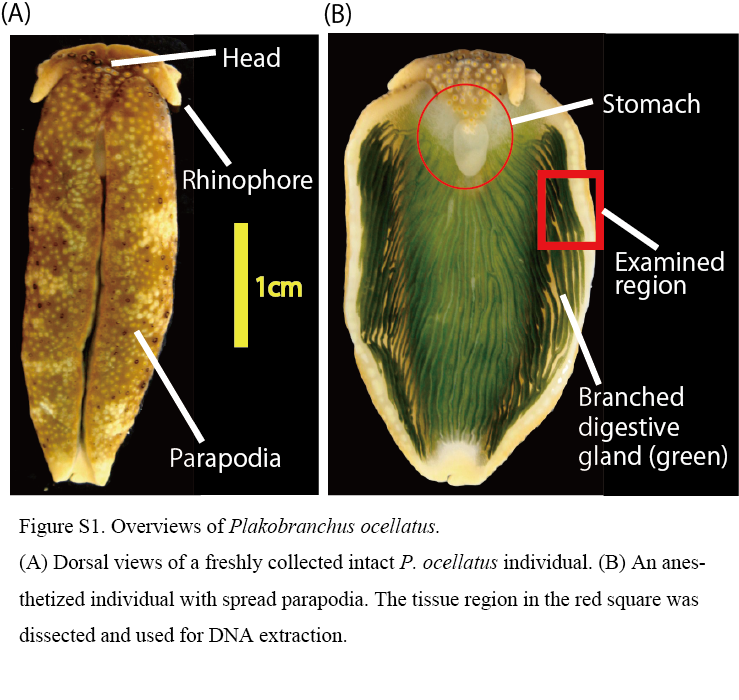

Supplement: Figure S1 — Overviews of Plankobranchus ocellatus . (A) Dorsal views of a freshly collected intact P. ocellatus individual. (B) An anesthetized individual with spread parapodia. The tissue region in the red square was dissected and used for DNA extraction. (TIF) [file pone.0042024.s001.tif]
